# Botanical and Genetic Identification Followed by Investigation of Chemical Composition and Biological Activities on the *Scabiosa atropurpurea* L. Stem from Tunisian Flora

**DOI:** 10.3390/molecules25215032

**Published:** 2020-10-29

**Authors:** Soukaina Hrichi, Raja Chaabane-Banaoues, Sihem Bayar, Guido Flamini, Yassine Oulad El Majdoub, Domenica Mangraviti, Luigi Mondello, Ridha El Mzoughi, Hamouda Babba, Zine Mighri, Francesco Cacciola

**Affiliations:** 1 Laboratory of Physico-Chemistry of Materials, Faculty of Sciences of Monastir, University of Monastir, Monastir 5000, Tunisia; soukaina.hrichi@gmail.com (S.H.); zinemighri@yahoo.fr (Z.M.); 2 Laboratory of Medical and Molecular Parasitology and Mycology (LP3M), Faculty of Pharmacy of Monastir, Department of Clinical Biology B, University of Monastir, Monastir 5000, Tunisia; raja.chaabane@laposte.net (R.C.-B.); hamouda.babba@gnet.tn (H.B.); 3 Laboratory of Analysis, Treatment and Valorization of Environmental Pollutants and Products, Faculty of Pharmacy, University of Monastir, Monastir 5000, Tunisia; sihem.bayar@yahoo.fr (S.B.); ridha.mzoughi@rns.tn (R.E.M.); 4 Dipartimento di Farmacia, Università di Pisa, 56126 Pisa, Italy; guido.flamini@unipi.it; 5 Centro Interdipartimentale di Ricerca “Nutraceutica e Alimentazione per la Salute” (NUTRAFOOD), Università di Pisa, 56122 Pisa, Italy; 6 Department of Chemical, Biological, Pharmaceutical and Environmental Sciences, University of Messina, 98168 Messina, Italy; youladelmajdoub@unime.it (Y.O.E.M.); dmangraviti@unime.it (D.M.); lmondello@unime.it (L.M.); 7 Chromaleont s.r.l., c/o Department of Chemical, Biological, Pharmaceutical and Environmental Sciences, University of Messina, 98168 Messina, Italy; 8 Department of Sciences and Technologies for Human and Environment, University Campus Bio-Medico of Rome, 00128 Rome, Italy; 9 BeSep s.r.l., c/o Department of Chemical, Biological, Pharmaceutical and Environmental Sciences, University of Messina, 98168 Messina, Italy; 10 Department of Biomedical, Dental, Morphological and Functional Imaging Sciences, University of Messina, 98125 Messina, Italy

**Keywords:** *Scabiosa atropurpurea* L., phenols, carotenoids, HPLC, GC, mass spectrometry, antioxidant, antibacterial, antifungal, allelopathic

## Abstract

Scarce information about the phenolic composition of *Scabiosa atropurpurea* L. is available, and no carotenoid compounds have been reported thus far. In this study the phenolic and carotenoid composition of this plant was both investigated and associated bioactivities were evaluated. Aiming to obtain extracts and volatile fractions of known medicinal plants to valorize them in the pharmaceutical or food industries, two techniques of extraction and five solvents were used to determine the biologically active compounds. Gas chromatography coupled to flame ionization and mass spectrometry and liquid chromatography coupled to photodiode array and atmospheric pressure chemical ionization/electrospray ionization mass spectrometry highlighted the presence of 15 volatiles, 19 phenolics, and 24 natural pigments in *Scabiosa atropurpurea* L. stem samples; among them, the most abundant were 1,8-cineole, chlorogenic acid, cynaroside, and lutein. Bioactivity was assessed by a set of in vitro tests checking for antioxidant, antibacterial, antifungal, and allelopathic (against *Brassica oleracea* L. and *Lens culinaris* Medik) effects. *Scabiosa atropurpurea* L. stem extracts presented a considerable antioxidant, antibacterial, and allelopathic potential, with less antifungal effectiveness. These results indicate that the volatile fractions and extracts from *S. atropurpurea* L. stem could be considered as a good source of bioactive agents, with possible applications in food-related, agriculture, and pharmaceutical fields. Genetic investigations showed 97% of similarity with *Scabiosa tschiliensis*, also called Japanese *Scabiosa.*

## 1. Introduction

Extracts and essential oils recovered from plants have been described as a good natural reservoir, harboring bioactive secondary metabolites, with wide use in cosmetic, food, and pharmaceutical industries. They present a diversity of chemical structures, which are unsurpassed by the synthetic libraries. Novel scientific trends imply the usage of natural products, such as essential oils, plant extracts, or pure compounds in medical therapies [1]. Lately, researchers became interested to highlight the chemical composition of several plants, used by our ancestors to treat diseases. Subsequently, many instrumental techniques have been developed to give prompt information, which is the main choice for profiling complexes, especially for extracts and essential oils. Among these techniques, liquid chromatography coupled to mass spectrometry (LC-MS) turned out to be the most selected technique to identify bioactive compounds of extracts, providing corresponding phenolic and pigment profiles. On the other hand, gas chromatography coupled to mass spectrometry (GC-MS) has been maintained to obtain the volatile profile of plants [2]. The bioactive compounds obtained from plants correspond to the major biochemical classes; among them, phenols, pigments, alkaloids, and terpenes possess very important biological activities, namely antioxidant, antifungal, antibacterial, anti-inflammatory, anticancer, antiviral, and allelopathic [3,4,5,6,7,8]. *Scabiosa* is a small genus of Dipsacaceae (Caprifoliaceae) family, represented by about 100 species all over the world and most of them grows in the Mediterranean region [9]. Eleven species of *Scabiosa* were identified from Tunisian flora: *Scabiosa arenaria* forssk, *Scabiosa stellata* L.; *Scabiosa crenata* Cyr.; *Scabiosa daucoides* Desf.; *Scabiosa robertii* Bonn, *Scabiosa atropurpurea* L. (*S. atropurpurea* L.), *Scabiosa rutifolia* Vahl.; *Scabiosa farinose* Coss.; *Scabiosa succisa* L.; *Scabiosa simplex* Desf.; and *Scabiosa thysdrusiana* [10]. Many sources indicated various biopotential and therapeutic benefits of *Scabiosa* species in the Mediterranean area. Accordingly, the aerial part of *S. columbaria* was used to treat diphtheria in Spain [11] and the flowers and the leaves of *Scabiosa stellata* L were used as a cracked heel remedy in Moroccan tradition [12]. Moreover, several biological proprieties, such as antibacterial [13,14,15] and antioxidant [16,17] activities, were accredited to *Scabiosa* species. The health benefits of *Scabiosa* are often attributed to its content in phenols [18] and iridoid glucosides [19]. *S. atropurpurea* L. is a perennial herb reaching 60 cm in size [20], distributed in the Mediterranean, Europe, Asia, and southern Africa [8]. The flowers of *S. atropurpurea* L. have been used as herbal tea to treat hypoglycemia [21], but also in several diseases, such as acne, bronchitis, cold, and cough, thanks to its analgesic, antipyretic, anti-inflammatory, and antibacterial activities. Aerial parts have been used as a veterinary diuretic (Iberia) and for menstrual regulation (Northern Peru) [22,23,24,25]. Scarce phytochemical profiling has been reported on *S. atropurpurea* L.; and only the roots and flowers of the plant have been subjected to silica gel column chromatography after extraction with methanol [19]. The pre-cited study described the iridoid glycosides as the main isolated compounds from *S. atropurpurea* L. In this study, for the first time the inspection of photochemical profiles on volatiles compounds, phenols, and carotenoids from Tunisian *S. atropurpurea* L. stems, using the GC-FID/MS, LC-PDA-ESI/MS, and LC-PDA-APCI/MS analyses, respectively, were investigated. Before these techniques could be applied, dry content was dissolved in distilled water to extract the essential oil, then fractioned with hexane and chloroform through a hydrodistillation process. Equally, the powder was dissolved successively in four solvents (dichloromethane, chloroform, ethyl acetate, and ethanol), to obtain four different extracts by hot extraction. 

The usefulness of the extracted liquid (hot extraction) and volatile (hydrodistillation) compounds in different biological activities were also assessed. 2,2-diphenyl-1-picrylhydrazyl (DPPH) radical scavenging method was used to determine their antioxidant potential. The antibacterial and antifungal capacities were evaluated by the microdilution method, against seven pathogenic microorganisms. Additionally, the study of the allelopathic effect of extracts against *Lens culinaris* Medik (lens) and *Brassica oleracea* L. (Kohlrabi) seeds, as a potential green eco-friendly biofertilizer or bioherbicide, was carried out.

## 2. Results and Discussion

### 2.1. Identification of Plant Material

The collected plant specimens from Kondar province were typically close to *S. atropurpurea* L. (Dipsacaceae), also named Sixalix Raf.; Fl. Tellur. (1838) and *Scabiosa thysdrusiana* (Le Houerou), an endemic plant from the Tunisian flora [26]. The anatomical properties and the shape of this species were carefully described along with measurements of stems, leaves, and roots [20]. Differently from other species, *S. atropurpurea* L. Greuter & Burdet, with its bluish-lilac flower color and the shape of its fruit, is a biennial or perennial plant which is 20–60 cm in height, spreading in circum-Mediterranean, sandy desert soils, rocky slopes, and meadows up to 2000 m [27].

**Figure 1:**
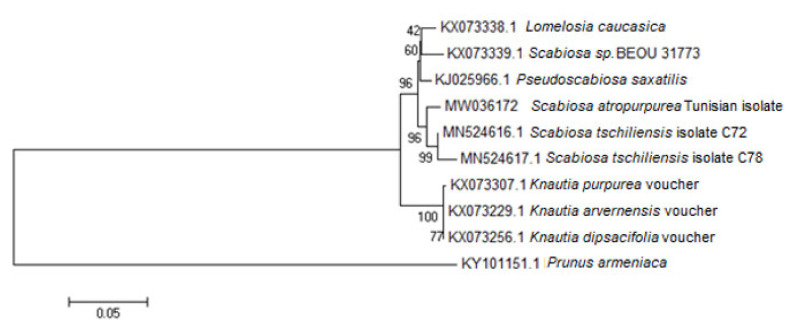
Neighbor-Joining (NJ) tree analysis of *Scabiosa* and closely related species inferred from the petN-psbM intergenic region was constructed using Mega, with *Prunus armeniaca* L. as Outgroup. Bootstrap value with 1000 replicates.

The genetic investigations based on the *petN-psbM* intergenic spacer region of the chloroplast DNA reinforced the membership of the studied plant to *Scabiosa* genus, with a difference from the *Knautia* genus supported by a 100% bootstrap value (Figure 1). The *S. atropurpurea* L. isolate, described herein, was clustered in the same node with *Scabiosa tschiliensis* (bootstrapping value = 96%) (Figure 1), allied taxa to *Scabiosa comosa* (Korea and China), and *Scabiosa japonica* (Japan) [28]. Phylogenetic relationships in Dipsacaceae inferred by DNA sequence data, conducted by using three chloroplast markers (atpB-rbcL, trnL-trnF, trnSVGA-trnGGCC), corroborated our findings that *Sixalix atropurpurea* and *Scabiosa japonica* are grouped in the same clade. 

### 2.2. Extraction of Second Metabolites

The means of three triplicate yields were calculated and values in percentage of total stem weight (wt%) are reported (Table 1). Hydrodistillation provided two yellow volatile fractions VF1 and VF2 with 0.032% ± 0.002 and 0.0122% ± 0.001 yields, referring to hexane and chloroform, respectively.

### 2.3. Identification of Volatile Compounds

The analysis of the composition of the volatile fractions (VF1 and VF2) recovered by hydrodistillation from *S. atropurpurea* L. stem showed different percentages in compounds, according to the solvents employed, namely hexane and chloroform. A total of 15 volatile compounds were isolated, identified, and quantified by GC-FID/MS in the VF1 and VF2 fractions, and accounted for 90.8% and 93.8% of total volatile constitutes (Table 2), respectively. A significant difference was observed for the contents in oxygenated monoterpenes, sesquiterpene hydrocarbons, phenylpropanoids, and other derivatives between VF1 and VF2. However, no significant difference was observed for the contents in apocarotenes. The most abundant volatile compounds detected in *S. atropurpurea* L. stem were 1.8 cineole, tetradecene, and (E)-*β*-ionone, with contents ranging from 8.1–33.5%, 5.7–24.1% and from 5.9–20.7%, respectively. In particular, dihydroactinidiolide was only detected in VF2 with a content equal to 26.1%. Most of these compounds were reported in essential oils extracted from *S. arenaria* [29] and *Scabiosa flavida* [30]. However, the GC-MS profiling of the *S. stellata* lipophilic extract from Algeria showed sitosterol, ursolic acid, and oleanolic acid as the most abundant terpenes [31]. 

Volatile and aromatic compounds extracted from medicinal and aromatic plants are known to have important biological effects. Oxygenated monoterpenes and sesquiterpenes hydrocarbons constitute a subclass of terpenes that have been known to show a wide range of biological activities, such as antimicrobial [32], anti-inflammatory [33], anticancer [34], and insecticidal activities [35].

### 2.4. Determination of the Phenolic Compounds in S. atropurpurea L. Stem

**Figure 2:**
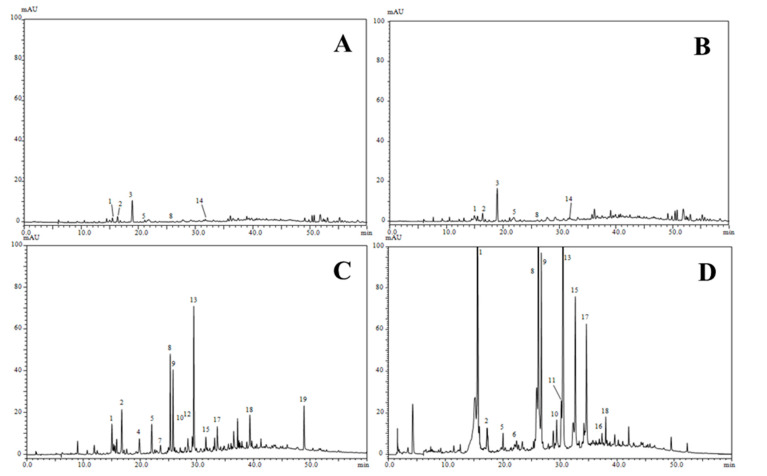
Phenolic profiles of *S. atropurpurea* L. stem extracts obtained by HPLC-PDA analysis, (**A**); dichloromethane extract, (**B**); chloroform extract, (**C**); ethyl acetate extract, (**D**); ethanol extract.

Identification of the phenolic compounds was carried out by the comparison of PDA absorption (λ_max_), the specific mass-to-charge ratio (*m/z*) with data reported from literature (Figure 2, Table 3). A total of 19 compounds were characterized by HPLC-PDA and HPLC-ESI/MS, and tentative identification is summarized in Table 3. These compounds belong to two different phenolic classes according to their chemical structure, including seven phenolic acids and seven flavonoids. A distribution that tracked the number of phenolic compounds in each sub-class revealed the presence of esters (chlorogenic acid), phenolic aldehyde (vanillin), hydroxycinnamic acid derivatives (caffeic acid, *p*-coumaric acid, *p*-hydroxycinnamic acid, and dicaffeoylquinic acid), flavones (cynaroside, hyperoside, luteolin hexoside, and luteolin), isoflavonoid (isoquercitrin), and lignin (syringaresinol hexoside) in *S. atropurpurea* L. stem extracts. Among the characterized molecules, all compounds detected in dichloromethane extract were also detected in chloroform extract, while only nine of them were common in the ethyl acetate and ethanol extracts. A total of four compounds were present in all extracts (chlorogenic acid, caffeic acid, *p*-hydroxycinnamic acid, and cynaroside). This difference might be explained by the difference in polarity between the solvents used in the extraction process. To the best of the authors’ knowledge, the profiling in phenolic compounds from *S. atropurpurea* L. stem extracts is herein reported for the first time. The phenolic concentration reached a maximum at 2790.74 μg per gram of ethanol extract, followed by ethyl acetate extract (199.31 μg g^−1^), the chloroform extract (201.83 μg g^−1^), and dichloromethane extract (129.15 μg g^−1^). Cynaroside was the most abundant phenolic compound in the range of 39.79–741.60 μg g^−1^, with the highest amount detected in the ethanol extract. Such a compound was reported as anti-inflammatory and anti-allergic agent [36] and it has been detected frequently in *S. atropurpurea* L. from Egypt, but also in other *Scabiosa* species (*S. olgae, S. tenuis,* and *S. argentea*) [18,37,38]. Chlorogenic acid was present in all extracts with a value ranging from 6.08 to 657.78 μg g^−1^, and relatively the highest amount was detected in ethanol extract. Chlorogenic acid is a dietary polyphenol known for its important biological activities [39] and it has been detected in *Scabiosa olgae* [38] and *Scabiosa bipinnata* [40]. Recently, HPLC analysis of phenolic compounds from *S. tschiliensis* whole plant demonstrated a high content in chlorogenic acid, especially in the ethyl acetate extract (45.35 ± 2.63 mg g^−1^). Caffeic acid and quercimeritrin were also common with this *Scabiosa* species [41].

Quantitative investigations demonstrated an important difference between polar and apolar extracts for vanillin. In apolar extracts (dichloromethane and chloroform extract), the vanillin was detected as the major compound, with a value of 104.11 μg g^−1^; nevertheless, it was not detected in polar extracts (ethyl acetate and ethanol extracts). Vanillin, a condensed tannin, is used as a food additive and its presence was previously highlighted in *Scabiosa* species; *S. hymettia* [14], *S. arenaria* [16], and *S. stellata* [31]. On the other hand, the flavones luteolin hexoside (564.58 μg g^−1^) and two dicaffeoylquinic isomers (224.38 and 213.63 μg g^−1^) were major acids detected exclusively in a polar extract with important values (Table 3). Luteolin, polyhydroxylated flavones, has been detected in ethanol extract from the Egyptian *S. atropurpurea* L. leaves and stems, with no quantitative data [18]. The same study revealed luteolin-7-O-gentiobioside, luteolin-7-O-D-rutinoside, and luteolin derivatives in methanol and butanol extracts. Additionally, luteolin was found to be ubiquitous in other *Scabiosa* species (*S. tenuis* and *S. stellata*) and identified in ethanol and methanol extracts [31,37]. A high dietary intake of luteolin seems to low the risk of acute myocardial infarction, and it was proven to have in vivo cancer chemopreventive properties [42]. The dicaffeoylquinic acid isomers detected herein indicating a molecular formula of C_25_H_24_O_12_ and showing a deprotonated ion peak at *m/z* 515, was closely related to the chlorogenic derivatives 3,5-O-dicaffeoylquinic and 4,5-O-dicaffeoylquinic acids, with the same formula and different protonation level (*m/z* 517), recently isolated from *S. stellata* [31]. The analgesic and anti-inflammatory effects of dicaffeoylquinic acids have also been reported [41].

### 2.5. Identification of the Pigment Compounds in S. atropurpurea L.

**Figure 3:**
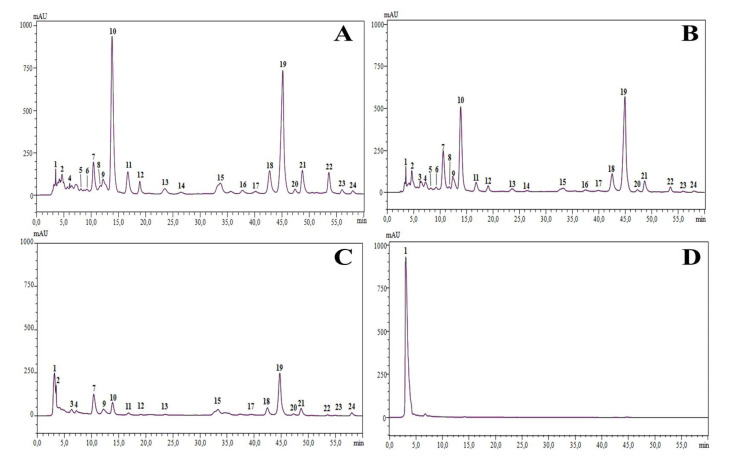
Pigment profiles of different extracts of *S*. *atropurpurea* L. stem obtained by HPLC-PDA analysis, (**A**); dichloromethane extract, (**B**); chloroform extract, (**C**); ethyl acetate extract, and (**D**); ethanol extract.

Principal biochemical classes of pigments identified in this study from *S. atropurpurea* L. stem were chlorophylls and carotenoids, all characterized by their importance for nurturing good health [43]. Twenty-four compounds were detected, 10 belonging to the family of chlorophyll and eight carotenoids, based on literature data (Figure 3, Table 4). The three following extracts, namely dichloromethane, chloroform, and ethyl acetate, have shown similar pigment profiles (Figure 3). However, different levels of carotenoid pigments were detected. The major detected compounds were lutein, most abundant in chloroform (247.57 µg g^−1^) and ethyl acetate (447 µg g^−1^) extracts, and 4,4-diapophytoene, which yielded 96.31 µg g^−1^ in the ethyl acetate extract (Table 5). Minor differences between these three extracts were observed for the rest of the compounds, such as actinioerythrin, isomer lutein and lutein. The ethanol extract presented a different pigment profile compared to other tested solvents, where only one carotenoid compound, 4,4-diapophytoene, was abundant (476.31 µg g^−1^). According to literature data, none of the *Scabiosa* species was characterized by its carotenoid composition.

### 2.6. Antioxidant Activity

**Figure 4:**
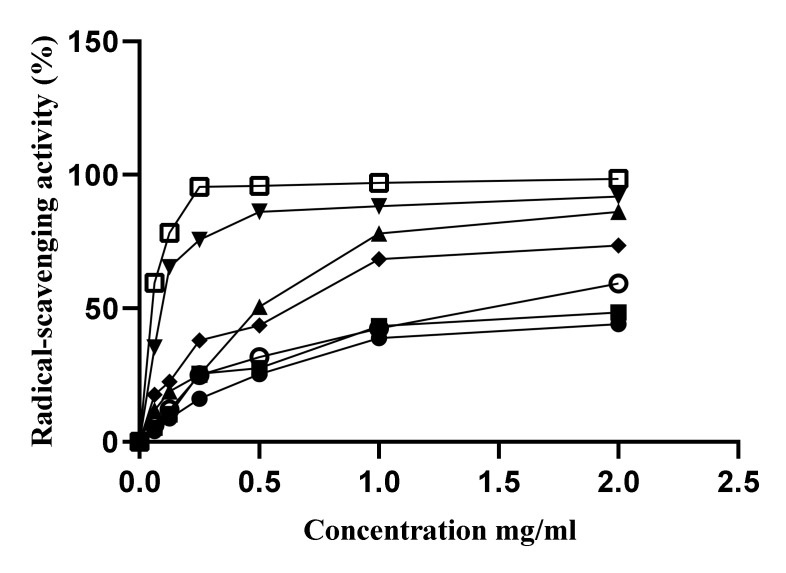
Antioxidant capacity of volatile and non-volatile metabolites of *S. atropurpurea* L stem; 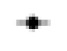 Dichloromethane extract; 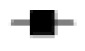 Chloroform extract; 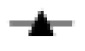 ethyl acetate extract; 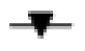 ethanol extract; 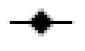 VF1; 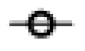 VF2; 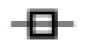 Ascorbic acid.

The free radical scavenging assay is widely used to evaluate the antioxidant capacity of different plant second metabolites. The free radical-scavenging activity of extracts and volatile fractions from *S. atropurpurea* L. stem was evaluated by the DPPH assay and compared to ascorbic acid (Figure 4, Table 6). Among all the extracts, ethanol extract had the highest antioxidant capacity (IC_50_, 0.1383 ± 0.0789 mg mL^−1^) followed by the ethyl acetate extract (IC_50_, 0.4806 ± 0.0487 mg mL^−1^). However, chloroform extract (IC_50_, 2.0951 ± 0.3750 mg mL^−1^) and dichloromethane extract (IC_50_, 2,6985 ± 0.4296 mg mL^−1^) exhibited weak scavenging activity. Volatile fractions acted as extracts, following the polarity gradient, with more effectiveness for polar factions. Thus, the hexanoic VF1 fraction (IC_50_, 0.4798 ± 0.0897 mg mL^−1^) presented a maximum of activity compared to the chloroformic VF2 fraction (IC_50_, 1.2944 ± 0.2067 mg mL^−1^). The antioxidant potential of second metabolites extracted from *S. atropurpurea* L. was dependent on the presence of different bioactive compounds, such as phenolic acids, flavonoids, carotenoids, and chlorophylls [44,45]. There is scarce information regarding the antioxidant activity of *S. atropurpurea* L. [18]*,* who studied the scavenging effect of the aerial parts from the Egyptian *S. atropurpurea* L.; concluding that the ethanol extract and ethyl acetate fraction expressed high capacity with 2.2% and 3.03% of change from control, respectively. The ethyl acetate fraction from the pre-flowering stage of *S. tschiliensis* Grunning appeared to contain the highest content of chlorogenic acid and demonstrated higher DPPH radical-scavenging activity with the IC_50_ value of 8.47 ± 0.23 µg mL^−1^, which was nearly equal to the IC_50_ value of vitamin C (7.60 ± 0.61 µg mL^−1^) [41].

### 2.7. Antibacterial and Antifungal Activities 

The antibacterial and antifungal activities of the extracts and volatiles fractions obtained from *S. atropurpurea* L. stem were evaluated against seven microorganisms including two Gram-negative bacteria, two Gram-positive bacteria, and three human pathogenic yeasts (*Candida* spp.). The highest antimicrobial activity was observed with the volatile fraction VF1 against the Gram-negative bacteria *Escherichia coli* with a minimum bactericide concentration (MBC) at 0.75 mg mL^−1^ followed by VF2 against *Salmonella enterica* with MBC at 0.87 mg mL^−1^ (Table 7). Only VF1 showed an antifungal effect on all tested strains with a minimum fungicide (MFC) at 1 mg mL^−1^. However, a moderate anti-microbial activity observed against the Gram-positive bacteria *Enterococcus faecalis* and *S. aureus* with MBC at 1.5 mg mL^−1^ for the volatile fraction VF1. Major compounds of VF1 were Tetradecene and (E)-*β*-ionone. *β*-ionone derived chalcones were described as potent antimicrobial agents [46]. Tetradecene was reported from Gynura segetum’s leaf extracts to be effective against a large panel of pathogenic bacteria [47]. 1,8-cineole [48], most abundant in VF2, was described to possesses an important antimicrobial effect against *S. aureus*, methicillin-resistant *S. aureus*, *Pseudomonas aeruginosa*, *E. coli*, *Klebsiella pneumoniae*, *Enterococcus faecalis,* and *C. albicans.*

### 2.8. Allelopathic Activity

**Figure 5:**
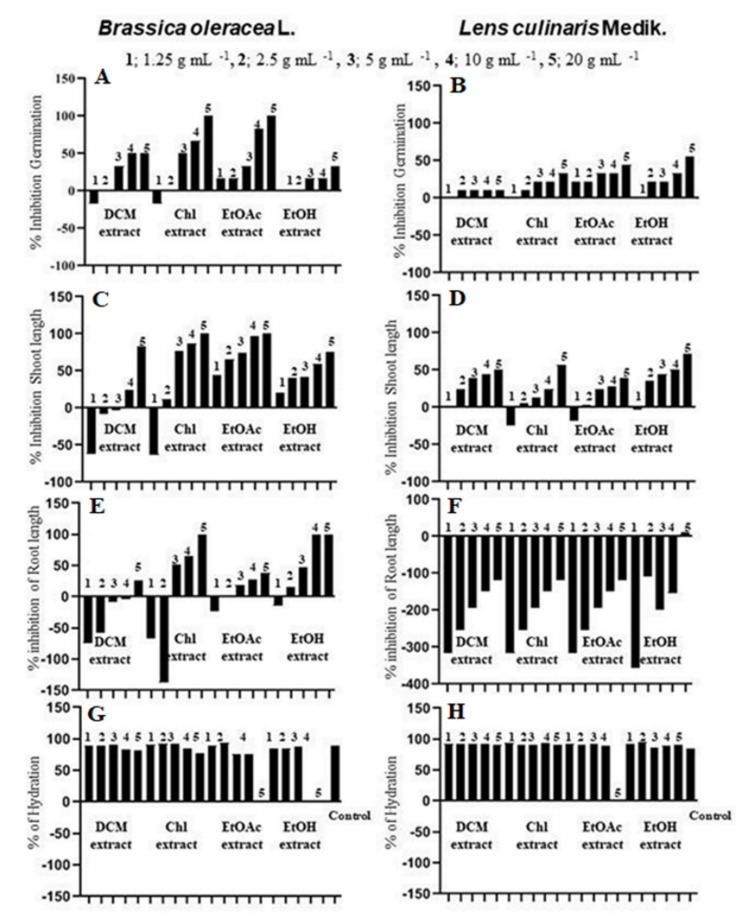
Allelopathic effects of dichloromethane (DCM), Chloroform (Chl), Ethyl acetate (EtOAc), and ethanol (EtOH) extracts from *S. atropurpurea* L. stem on the germination (**A**,**B**), shoot length (**C**,**D**), root length (**E**,**F**), and hydration (**G**,**H**) of *Lens culinaris* Medik. (lens) and *Brassica oleracea* gongylodes (kohlrabi). Significant difference, one-way ANOVA, *p* < 0.05.

The seed germination, seedling growth and hydration of *Brassica oleracea* L. (kohlorobi) and *lens culinaris* Medik (lentils) were obtained upon exposure to *S. atropurpurea* L. stem dichloromethane, chloroform, ethyl acetate, and ethanol extracts at five different concentrations (Figure 5). For germination, water control showed 60% and 90% germination in kohlrabi and lentils seeds, respectively.The organic extracts of *S. atropurpurea* L. stem showed different percentages of seed germination inhibition, which decreased as the concentration decreased. A complete failure of germination was recorded in chloroform and ethyl acetate extracts at a concentration of 20 mg mL^−1^, while a stimulation of germination of kohlrabi was noticed in dichloromethane and ethanol extracts at 1.25 mg mL^−1^. Moreover, the germination of lentil species was moderately inhibited by all extracts. The inhibition percentage on seed seedling differed among extracts and the species. Indeed, kohlrabi shoot and root lengths were reduced at the highest concentration of each extract (20 mg mL^−1^). For lentils, the shoot length was reduced at 20 mg mL^−1^ with percentages ranging from 2.71% to 71% and stimulated at lower concentrations with a percentage range from −24.63% to 2.71%. In contrast, the root lengths were stimulated with a percentage from −357% to −5%. Compared to control, the hydration effect on seedling was similar. The attained results demonstrate the allelopathic effect of *S*. *atropurpurea* L. stems, and this effect depended on the quality and quantity of allelochemical compounds, e.g.; phenolic compounds, which are reported as herbicides or pesticides according to their structure [49], namely chlorogenic acid, caffeic acid derivatives [50], *p*-coumaric acid, cinnamic acid, and vanillin [51]. Such results might support the use of *S. atropurpurea* L. extracts as potential allelopathic substances, which would be tested as a potential natural herbicide resource. 

## 3. Materials and Methods 

### 3.1. Sample Collection and Identification of Plant Material 

The fresh stems of annual *Scabiosa* speeding in the province of Kondar (35°55′58′′ N latitude, 10°18′00′′ E longitude) from the Tunisian Sahel, situated about 30 km from the north-west of Sousse governorate, were harvested during May 2017, when the plant was flowering. The species identification was based on shape and morphological features of *Scabiosa* leaves, flowers, and fruits, compared to scientific botanic illustrations [22,29,30]. Moreover, a genetic characterization using bio-molecular tools were applied to plant fresh material in order to confirm genus and species identification. Briefly, DNA from *Scabiosa* stems, freshly collected was extracted using the chelating resin Chelex 100 [52]. The extraction eluate was subjected to a polymerase chain reaction (PCR) amplification of a Chloroplastic DNA fragment, using the species- and group-specific primers “ycf6F” and “psbMR”, targeting for the *petN-psbM* intergenic spacer region [53]. The PCR products were purified and sequenced. The sequence alignment was performed at first with BLAST (Basic Local Alignment Search Tool) algorithm [54]. Then, the obtained sequence (MW036172) was aligned with reference sequences of *Lomelosia caucasica* (KX073338.1), *Scabiosa* sp. (KX073339.1), *Scabiosa tschiliensis* (MN524616.1; MN524617.1), *Pseudo scabiosa saxatilis* (KJ025966.1), *Knautia purpurea* (KX073307.1), *Knautia arvensis* (KX073229.1), and *Knautia dipsacifolia* (KX073256.1) from the NCBI database. Genetic distances computing and phylogenetic trees inferring were realized using MEGA 5.0 software-based on Neighbor-Joining (NJ) method, with *Prunus armenica* L. (KY101151.1) as outgroup.

### 3.2. Chemicals and Reagents

All solvents used in the experiments (hexane, dichloromethane, chloroform, ethyl acetate, and ethanol) were analytical grade (Merck Life Science, Merck KGaA, Darmstadt, Germany). LC-MS grade methanol, acetonitrile, acetic acid, and water were obtained from Merck Life Science (Merck KGaA, Darmstadt, Germany). The employed phenolic standards used for the quantification were five, namely chlorogenic acid, gallic acid, caffeic acid, coumarin, and rutin, all obtained from Merck Life Science (Merck KGaA, Darmstadt, Germany). Stock solutions of 1000 mg L^−1^ were prepared for each standard by dissolving 10 mg in 10 mL of methanol. Dimethyl sulfoxide (DMSO) was purchased from BIO BASIC INC (Desk, Canada). Culture media were purchased from Sigma-Aldrich (CHEMIE GmBH, Riedstr, Germany). RPMI-1640 medium was purchased from Gibco and stored at 4 °C. 

### 3.3. Preparation of Plant Volatile Fractions and Extracts from Stems of S. atropurpurea L.

The plant material was air-dried at room temperature in the shadow and ground to a fine powder, later used for extractions. In order to extract volatile compounds, 150 g of the plant powder were subjected to hydro-distillation for 3 h, after liquid-liquid extraction with hexane and chloroform, successively. The two volatile fractions VF1 (hexane) and VF2 (chloroform) were stored at 4 °C in sealed brown glass vials until uses.

The whole process from preparing the dichloromethane extract from stem powder to the ethanol extract was following the protocols detailed by Hrichi et al. [55]. Briefly, 100 g of stem powder was subject to four successive extractions, using for each 300 mL of solvent (dichloromethane, chloroform, ethyl acetate, and ethanol), after boiling for 90 min, filtrating with filter paper, the solvent was evaporated from the filtrate and the residue was used for the next extraction. The resulting extract of each extraction was stored until use at 4 °C.

### 3.4. Analysis of Volatile Compounds

Separation and quantification of volatile compounds of the fractions VF1 and VF2 were obtained after the hydrodistillation process was performed on HP-5890 Series II instruments, equipped with HP-WAX and HP-5 capillary columns (30 m × 0.25 mm, 0.25 μm film thickness), with an oven temperature program as follows: 60 °C for 10 min, the ramp of 5 °C min^−1^ up to 220 °C; injector and detector temperatures 250 °C; carrier gas was helium (2 mL min^−1^); detector dual Flame Ionization Detector (FID); split ratio of 1:30; injection of 0.5 μL (10% hexane solution). The identification of components with GC-FID was by comparing their linear retention index (LRI), relative to the series of *n*-hydrocarbons. The GC-MS analyses were performed on a Varian CP-3800 gas-chromatograph equipped with an HP-5 capillary column (30 m × 0.25 mm; coating thickness, 0.25 μm) and a Varian Saturn 2000 ion trap mass detector. The analytical conditions were as follows: injector and transfer line temperatures 220 and 240 °C, respectively; oven temperature programmed from 60 to 240 °C at 3 °C/min; carrier gas helium at 1 mL min^−1^; injection of 0.2 μL (10% hexane solution); split ratio of 1:30. Constituents identification was based mainly on a comparison of their linear retention indices relative to the series of *n*-hydrocarbons (C7–C28) and computer matching against the commercial and homemade libraries of mass spectra, and MS literature data [56,57]. Quantitative results were expressed as a percentage of total volatile compounds. 

### 3.5. Analysis of Phenolic Compounds 

The phenolic analysis of the dichloromethane, chloroform, ethyl acetate, and ethanol extracts of *S. atropurpurea* L. stem (identification and quantification) followed the methodology described by Hrichi et al. [55] and Haoujar et al. [58]. The phenolic compounds were determined using a high performance liquid chromatography system coupled to photodiode array and mass spectrometry detection (LC-PDA- ESI/MS). 

### 3.6. Analysis of Carotenoids 

In order to determine the content of dichloromethane, chloroform, ethyl acetate, and ethanol extracts of *S. atropurpurea* L. stem in carotenoids, 100 mg of each extract were dissolved in 1 mL of methanol/methyl terbutyl ether (*v*/*v*) mixture solution. The obtained solutions were filtered through a 0.45 µm Acro-Disc nylon membrane (Merck Life Science, Merck KGaA, Darmstadt, Germany) before to be analyzed by LC-PDA-APCI/MS. The procedure of the identification and quantification was carried out using the analytical methodology reported by Hrichi et al. [55].

### 3.7. Antioxidant Activity

DPPH radical scavenging assay was adopted to measure the ability of biosynthesized compounds extracted from *S. atropurpurea* L. stem to scavenge free radicals. The activities were evaluated according to the method described by Hrichi et al. [55], with a few modifications. Serial dilutions in ethanol were prepared to obtain six concentrations (2, 1, 0.5, 0.25, 0.125, and 0.0625 mg mL^−1^) of each extract, volatile fraction, and ascorbic acid (standard). A 0.1 mM DPPH solution was prepared in ethanol. A 0.5 mL of each diluted sample was added glass vessel followed by the introduction of 0.5 mL of DPPH solution. After 30 min of incubation in obscurity at room temperature, the absorbance was measured at λ = 517 nm. Ethanol was used as the blank solution; a control was DPPH/ethanol (*v*/*v*) solution. The percentage of scavenging free radicals by the tested samples was calculated with Equation (1):

% SFR = ((A _control_ − A _sample_)/A _control_) × 100 (1)

where A _control_ is the absorbance of the control sample and A _sample_ is the absorbance of the tested sample. The IC_50_ is defined as the occurrence of 50% of radical scavenging by each sample.

### 3.8. Antibacterial and Antifungal Activities

Microorganisms used for this study were selected as follow: three yeast strains (*Candida albicans* ATCC 90028, *Candida tropicalis* ATCC 66029, and *Candida glabrata* ATCC 64677), two Gram-positive bacteria (*Staphylococeus aureus* ATCC 25923 and *Salmonella Typhimurium* ATCC 1408) and two Gram-negative bacteria (*Escherichia coli* ATCC 35218 and *Enterococus feacolis* ATCC 29212). Cultures for antibacterial and antifungal tests were grown on Mueller-Hinton and Sabouraud chloramphenicol plates, respectively, prepared from commercial powder, at 37 °C for 24 h. The minimum inhibitory concentrations (MICs) of *S. atropurpurea* L. stem extracts and volatile fractions were determined in RPMI 1640 medium solution (with L-glutamine/without sodium bicarbonate) supplemented with glucose (2%), using the broth microdilution method in a 96-well and using a colorimetric assay with resazurin redox indicator for the viability testing. Each sample was dissolved in 10% DMSO to obtain a primary concentration of 200 mg mL^−1^, and then a serial two-fold dilution of the extracts and volatile fractions was realized. Overnight cultures of strains were inoculated to yield a final concentration of 10^5^ CFU. Standard drugs, Amphotericine b and Gentamicin, were used as positive references. The MICs were considered as the lowest concentration giving an inhibition of the pathogen strains. The MBCs and MFCs concentrations were determined by seeding 10 µL from all MICs wells onto Muller-Hinton and Sabouraud chloramphenicol plates, respectively, and incubated at 37 °C for 24 to 48 h. Data from three replicates were evaluated and modal results were calculated.

### 3.9. Allelopathic Activities 

The activity of dichloromethane, chloroform, ethyl acetate, and ethanol extracts on the germination, root, and shoot length elongation and hydration of *Lens culinaris Medik*. (lens) and *Brassica oleracea* L. (kohlrabi) species seeds were evaluated according to the method described by Teerarak et al. [59], with a few modifications. Five concentrations (8, 4, 2, 1, and 0.5 mg mL^−1^) were prepared for each tested extract, compared to a control (distilled water). Briefly, 1 mL of each concentration sample was piped to a filter paper discs (Whatman ™, 9 mm), placed in a 9 cm diameter petri dish. The solution was allowed to evaporate to air for 24 h, and then 5 mL of distilled water were poured onto each filter paper disc. Ten healthy seeds of each species, previously soaked in distilled water for 2 h, were placed on the filter paper disc. All tests were done in triplicates. Petri dishes were left under ambient temperature, light and humidity conditions in the laboratory. The data to seed germination, shoot length, root length, and hydration were recorded after 7 days of sowing. The germination percentage was determined using Equation (2):

% G = [(NSS/TNS) × 100](2)

where NSS is the number of sprouted seeds and TNS is the total number of seeds. Germination inhibition percentages were calculated using Equation (3):

% I = 100 − % G(3)

The lengths of roots and shoots are measured and expressed in centimeters (cm). The percentages of elongation were calculated using Equation (4):

% I = [1 − (E/T)] × 100(4)

where E is the value of the parameter studied (length of the aerial part, length of the root part) in the presence of the extract and T is the value of the parameter studied (length of the aerial part, length of the root part) in the presence of the control (distilled water). The percentage of hydration was evaluated using Equation (5):

% H = [(PF − PS)/PF]*100(5)

where PF is the fresh sample weight and PS is the dry sample weight after drying at 60 °C for 24 h. The percentage of inhibition of hydration was calculated according to Equation (6):

% I = 100 − % H(6)

if % I > 0 there is inhibition; if % I < 0 there is stimulation.

### 3.10. Statistical Analysis 

Results of the effect of volatile fractions and extracts as allelopathic, antibacterial, antifungal, and antioxidant agents were reported as percentages of inhibition. The significance of differences among the various treatments was assessed by using the one-way analysis of variance (ANOVA), followed by Tukey’s post hoc test. IC_50_ values for DPPH radical scavenging method were calculated. Values of *p* < 0.05 were considered as significantly different. Statistical analysis was carried out using GraphPad Prism version 8. 0. software (GraphPad Software Inc.; La Jolla, CA).

## 4. Conclusions

In this study, the chemical profile and biological activities of the two volatile fractions and the four extracts from *S. atropurpurea* L. stem are reported for the first time. The volatile chemical profile revealed that the most abundant compounds were 1,8-cineole, dihydroactinidiolide, tetradecene, and E-*β*-ionone. The non-volatile chemical profiles revealed that the most abundant phenolic compounds were cynaroside and chlorogenic acid and the most encountered carotenoid was lutein. The ethanol extract yielded the highest number of phenolic compounds, while the dichloromethane extract yielded the highest number of pigment compounds. *S. atropurpurea* L. stem proved to be a rich source of antioxidant, antibacterial, and allelopathic substances with great health-promoting potential, and could be utilized in agriculture and pharmaceutical industries. 

## Figures and Tables

**Table 1:** *S. atropurpurea* L. stem volatile fraction and extract yield using different solvents.

Name	Extraction Method	Solvent	wt% ± SD
**VF1**	Hydro-distillation	Hexane	0.032 ± 0.002
**VF2**	Hydro-distillation	Chloroform	0.012 ± 0.001
**E1**	Hot-extraction	Dichloromethane	1.036 ± 0.021
**E2**	Hot-extraction	Chloroform	0.972 ± 0.008
**E3**	Hot-extraction	Ethyl acetate	0.834 ± 0.015
**E4**	Hot-extraction	Ethanol	1.132 ± 0.062

wt% ± SD: percentage ± standard deviation.

**Table 2:** Chemical composition of volatile fractions from *S. atropurpurea* L. stem, identified by GC-FID/MS.

Compound	L.R.I.	Concentration (%)	
		VF1	VF2
1,8-cineole	1034	8.1	33.8
*cis*-linalool oxide (furanoid)	1076	1.6	3.0
*trans*-linalool oxide (furanoid)	1090	n.d.	2.4
Linalool	1101	4.9	3.3
α-terpineol	1191	2.3	n.d.
Dihydrolinalylacetate	1275	n.d.	2.5
2-hydroxy-5-methylacetophenone	1315	4.4	2.9
Eugenol	1358	3.6	n.d.
*(E)-*β-damascenone	1382	6.4	3.0
**Tetradecene**	**1393**	**24.1**	5.7
*(Z)*-jasmone	1395	5.6	n.d.
β-caryophyllene	1419	n.d.	2.2
*(E)*-geranylacetone	1455	9.1	3.0
***(E)-*β-ionone**	**1488**	**20.7**	5.9
**Dihydroactinidiolide**	**1536**	n.d.	**26.1**
Oxygenated monoterpenes	-	16.9	45.0
Sesquiterpene hydrocarbons	-	0.0	2.2
Apocarotenes	-	36.2	38.0
Phenyl propanoids	-	3.6	0.0
Other derivatives	-	34.1	8.6
% peaks identified	-	90.8	93.8
Total yield% (mg 100 g^−1^)	-	0.032	0.012

The dominant compounds are indicated in bold. L.R.I.: linear retention index reported in the literature using the same GC stationary phase. VF1; volatile fraction extracted by hexane, VF2; volatile fraction extracted by chloroform. n.d.: not detected.

**Table 3:** Identification and quantification of the phenolic compounds in *S. atropurpurea* L. stem extracts by HPLC-PDA-ESI/MS.

Peak No	Rt (min)	λ_max_ (nm)	[M − H]^−^ *m/z*	Identification	Formula	Quantification (µg g^−1^ Extract (ppm)				Ref
						DCM	Chl	EtOAc	EtOH	
1	15.56	243, 325	353, 191	**Chlorogenic acid**	C_16_H_18_O_9_	14.81	6.08	87.99	**657.78**	[1,2,3]
2	16.73	240, 321	179	Caffeic acid	C_9_H_8_O_4_	2.57	5.92	130.16	45.98	[4]
3	19.42	208, 227, 279, 309	151	Vanillin	C_8_H_8_O_3_	64.67	104.11	n.d.	n.d.	[5]
4	19.86	235, 279, 375	163	*p*-coumaric acid	C_9_H_8_O_3_	n.d.	n.d.	16.49	n.d.	[6,7]
5	21.98	235, 309	163	*p*-hydroxycinnamic acid	C_9_H_8_O_3_	4.37	14.50	24.23	8.33	[8]
6	22.43	212, 270, 336	609, 367, 179	Unknown	-	n.d.	n.d.	n.d.	-	-
7	23.52	237, 285	485, 453, 403	Unknown	-	n.d.	n.d.	-	n.d.	-
8	25.45	231, 258, 268, 349	285, 447	**Cynaroside**	C_21_H_20_O_11_	39.79	71.22	199.97	**741.60**	[8]
9	25.98	243,348	464	Isoquercitrin	C_21_H_20_O_12_	n.d.	n.d.	108.72	205.57	[8]
10	28.59	240, 340	431	Hyperoside	C_21_H_20_O_12_	n.d.	n.d.	37.58	36.13	[8]
11	28.88	214, 273, 339	579, 455	Unknown	-	n.d.	n.d.	n.d.	-	
12	29.36	254, 345	463	Quercimeritrin	C_21_H_20_O_12_	n.d.	n.d.	36.05	81.36	[8]
13	29.64	253, 347	447	**Luteolin-hexoside**	C_21_H_20_O_11_	n.d.	n.d.	467.06	**564.58**	[8]
14	32.57	269	579	Syringaresinol hexoside	C_28_H_36_O_13_	LOQ	LOQ	n.d.	n.d.	[9]
15	32.68	215, 326	515	Dicaffeoylquinic acid isomer 1	C_25_H_24_O_12_	n.d.	n.d.	46.18	224.38	[4,8]
16	34.21	214, 335	615, 555, 447	Unknown	-	n.d.	n.d.	n.d.	-	-
17	34.65	218, 327	515	Dicaffeoylquinic acid isomer 2	C_25_H_24_O_12_	n.d.	n.d.	31.22	213.63	[4,8]
18	39.51	241, 347	285	Luteolin	C_15_H_10_O_6_	n.d.	n.d.	113.66	11.14	[7,8]
19	49.15	242, 268, 334	537, 329, 141	Unknown	-	n.d.	n.d.	-	n.d.	-
Total of phenolic compounds (µg g^−1^)						129.15	201.83	1299.31	2790.47	

DCM; Dichloromethane extract, Chl; Chloroform extract, EtOAc; Ethyl acetate extract, EtOH; Ethanol extract. The dominant compounds are indicated in bold. n.d.: not detected. LOQ, the limit of quantification. R_t_: retention time; [M + H]^+^: protonated molecule; λ_max_: ultraviolet absorption maxima.

**Table 4:** Pigment compounds identified in the extracts from *S. atropurpurea* L. stem by HPLC-PDA-APCI/MS.

N°	R_t_ (min)	λ _max_(nm)	[M + H]^+^ *m/z*	[M − H]^-^ *m/z*	Compounds	Formula	Ref
1	3.3	268	409	-	4,4′-diapophytoene	C_30_H_48_	[10]
2	3.5	530, 604, 658,	617	-	Chlorophyllide a	C_35_H_34_MgN_4_O_5_	[11]
3	6.4	281, 314, 421,434, 658	613	-	Chlorophyll c	C_35_H_32_MgN_4_O_5_	-
4	7.3	229, 279, 407, 504, 667	696, 609	712, 607	Unknown	-	-
5	8.1	238, 266, 401, 498, 667	625	-	Actinioerythrin	C_40_H_48_O_6_	[12]
6	9.05	232, 322, 407, 504, 666	637, 619	635, 389	Unknown	-	-
7	10.6	232, 331, 372, 437, 657	607	605	Pheophorbide b	C_35_H_34_ N_4_O_6_	[11,13]
8	11.6	415, 437, 464	551	568	Isomer lutein	C_40_H_56_O_2_	[14]
9	12.3	274, 340, 425, 507, 658	593	592	Pheophorbide a	C_35_H_36_N_4_O_5_	[15]
10	14.0	422, 444, 473	551	568	Lutein	C_40_H_56_O_2_	[16]
11	16.7	231, 409, 448, 467, 668	622	620	Unknown	-	-
12	19.0	416, 438, 467,	551	-	Echinenone	C_40_H_54_O	[15]
13	23.5	233, 269, 415, 438, 468	873, 765, 654	763, 652	Unknown	-	-
14	26.4	409, 507, 667	535	534	Torulene	C_40_H_54_	[17]
15	33.5	297, 326, 370, 436, 661	893	-	Chlorophyll a	C_55_H_72_MgN_4_O_5_	[11]
16	37.7	227, 279, 407, 504, 667	908, 887, 682	886, 680	Chlorophyll b	C_55_H_70_MgN_4_O_6_	[11,13]
18 17	39.9	282, 279, 407, 499, 504, 667	903	902	Unknown	-	-
18	43.14	331, 373, 437, 529, 661	885, 827	884, 826	Pheophytin b	C_55_H_72_N_4_O_6_	[11]
19	45.77	331, 371, 432, 441, 657	885, 827	884, 826	Pheophytin b	C_55_H_72_N_4_O_6_	[11]
20	47.5	276, 408, 507, 538, 667	-	870	Pheophytin a	C_55_H_74_N_4_O_5_	[11]
21	48.9	275, 340, 425, 507, 658	872	870	Pheophytin a	C_55_H_74_N_4_O_5_	[11]
22	53.4	428, 452, 478	537	-	*β*-carotene	C_40_H_56_	[15,18]
23	55. 8	417, 444, 472	537	-	9-*Z-β* -carotene	C_40_H_56_	[15,18]
24	58.15	412, 436, 507, 544, 663	827	826	Unknown	-	-

R_t_: retention time; [M + H]^+^: protonated molecule; λ_max_: ultraviolet absorption maxima.

**Table 5:** Quantification of carotenoid compounds in stem extracts of the species *S. atropurpurea* L.

Compounds	µg g^−1^Extract (ppm)			
	Dichloromethane	Chloroform	Ethyl Acetate	Ethanol
**4,4′-diapophytoene**	**19.75**	**19.45**	**96.31**	**476.31**
Actinioerythrin	18.85	14.28	n.d.	n.d.
Isomer lutein	27.55	16.78	n.d.	n.d.
**Lutein**	**447.74**	**247.58**	**35.77**	n.d.
Echinenone	32.20	17.90	3.90	n.d.
Torulene	11.22	6.70	n.d.	n.d.
***β*-carotene**	**64.35**	**14.63**	**4.56**	n.d.
9-*Z-β* –carotene	15.52	5.61	2.55	n.d.
Total	637.19	342.93	143.10	476.31

n.d.: not detected. The dominant compounds are indicated in bold.

**Table 6:** The relative antioxidant ability of different extracts and the volatile fractions of *S. atropurpurea* L. to reduce the half-maximal inhibitory concentrations (IC_50_) in the DPPH radical-scavenging activity assay.

Sample	IC_50_ mg mL^−1^
Dichloromethane extract	2.7085 ± 0.4296
Chloroform extract	2.0951 ± 0.3750
Ethyl acetate extract	0.4806 ± 0.0487
Ethanol extract	0.1383 ± 0.0789
VF 1	0.4798 ± 0.0897
VF 2	1.2944 ± 0.2067
Ascorbic acid	0.0840 ± 0.0103

**Table 7:** MICs and MBC/MFCs values (mg mL^−1^) of extracts and volatile fractions of *S. atropurpurea* L. stem on fungal and bacterial agents.

Test Sample and Standard	Gram-Negative Bacteria				Gram-Positive Bacteria				Yeasts					
	*S. enteric*		*E. coli*		*S. aureus*		*E. faecalis*		*C. albicans*		*C. tropicalis*		*C. glabrata*	
	MIC	MBC	MIC	MBC	MIC	MBC	MIC	MBC	MIC	MFC	MIC	MFC	MIC	MFC
E1	50	N.A.	0.78	1.56	50	N.A.	50	N.A.	N.A.	N.A.	N.A.	N.A.	N.A.	N.A.
E2	12.5	50	0.78	3.12	N.A.	N.A.	50	N.A.	N.A.	N.A.	N.A.	N.A.	N.A.	N.A.
E3	25	N.A.	12.25	25	50	N.A.	N.A.	N.A.	50	N.A.	N.A.	N.A.	N.A.	N.A.
E4	50	N.A.	25	N.A.	N.A.	N.A.	N.A.	N.A.	6.25	25	N.A.	N.A.	N.A.	N.A.
VF1	1.5	N.A.	0.75	1.5	N.A.	N.A.	3	3	1	1	1	1	1	1
VF2	0.22	0.87	1.5	N.A.	3.5	3.5	1.75	N.A.	N.A.	N.A.	N.A.	N.A.	N.A.	N.A.
Gentamicin	0.004	0.256	0.128	0.128	0.002	0.128	0.256	0.512	-	-	-	-	-	-
Amphotericine B	-	-	-	-	-	-	-	-	0.005	0.005	0.005	0.005	0.0025	0.0025

N.A.: non-active.
